# Low empowerment and diabetes regimen distress are related to HbA1c in low income type 1 diabetes patients in a Brazilian tertiary public hospital

**DOI:** 10.1186/s13098-019-0404-3

**Published:** 2019-01-22

**Authors:** M. S. V. M. Silveira, A. Moura Neto, A. C. Sposito, L. Siminerio, E. J. Pavin

**Affiliations:** 1 Internal Medicine Postgraduate Program, Faculty of Medical Sciences, State University of Campinas, Campinas, São Paulo Brazil; 2 Endocrinology Division, Department of Internal Medicine, Faculty of Medical Sciences, State University of Campinas, Campinas, São Paulo Brazil; 3 Cardiology Division, Department of Internal Medicine, Faculty of Medical Sciences, State University of Campinas, Campinas, São Paulo Brazil; 4 Diabetes Division, University of Pittsburgh, Pittsburgh, PA USA; 5 University of Pittsburgh, Pittsburgh, PA USA

**Keywords:** Diabetes mellitus, Type 1, Depression, Emotional distress, Empowerment, Patient

## Abstract

**Background:**

Adults with type 1 diabetes (T1D) have a high risk of developing depressive symptoms and diabetes-related distress (DD). Low socioeconomic level is associated with increased risk of poor self-management, treatment difficulties and psychological distress. The goals of this study were to document the frequency of major depressive disorder (MDD), high depressive symptoms and high DD, to assess levels of empowerment and to determine the association with each of these measures and glycemic control in a low-income Brazilian sample of adults with T1D.

**Methods:**

In a cross-sectional study, inclusion criteria were age > 18 years and diagnosis of T1D > 6 months. Exclusion criteria were cognitive impairment, history of major psychiatric disorders, severe diabetes-related complications and pregnancy. Diagnoses of MDD were made using interview-based DSM-5 criteria. Depressive symptoms were evaluated by the depression subscale of the Hospital Anxiety and Depression Scale (HAD-D). The Diabetes Distress Scale (DDS) assessed DD. Empowerment levels were evaluated by the Diabetes Empowerment Scale short form (DES-SF). Glycemic control was measured by HbA1c. The latest lipid panel results were recorded. Number of complications was obtained from medical records.

**Results:**

Of the 63 T1D patients recruited, 36.5% were male, mean age was 31.5 (± 8.9), mean number of complications was 1 (± 1.1), and mean HbA1c was 10.0% (± 2). Frequency of MDD was 34.9% and 34.9% reported high depressive symptoms. Fifty-seven percent reported clinically meaningful DD. High diabetes regimen distress and low empowerment were associated to HbA1c (*p* = *0.003*; *p* = *0.01*, respectively). In multivariate analyses, lower empowerment levels were associated to higher HbA1c (*beta* − *1.11*; *r-partial 0.09*; *p value 0.0126*). MDD and depressive symptoms were not significantly correlated with HbA1c in this expected direction (p = 0.72; p = 0.97, respectively).

**Conclusions:**

This study showed high rates of MDD, high depressive symptoms and high DD and low levels of empowerment in this low income population. Empowerment and diabetes regimen distress were linked to glycemic control. The results emphasize the need to incorporate the psychological and psychosocial side of diabetes into strategies of care and education for T1D patients.

## Background

Abundant evidence has shown that adults with type 1 diabetes (T1D) have a high risk for developing clinical depression and elevated depressive symptoms [1–5]. New research shows that over 40% of T1Ds experience elevated diabetes-related distress (DD) [6]. In order to maintain their glycemia at target levels, T1D patients need to monitor their blood glucose levels frequently and regularly, calculate and administer multiple daily insulin doses and address the effects of carbohydrate intake and exercise [7–9]. Living with T1D, the exhaustive demands of daily self-management and dealing with the possibility of developing chronic complications are associated with an increased risk of psychological distress [10, 11].

For patients with low incomes, the demands of managing T1D are even more challenging. These patients frequently do not have access to modern tools, diabetes education and other on-going support to assist them with disease management and to facilitate adaptive integration of their disease into every day life. Thus, low socioeconomic level is significantly associated with increased risk of poor self-management, treatment difficulties and psychological problems [12–14].

Diabetes education and psychosocial assessment are recommended as a critical part of patient-centered care in order to promote better diabetes outcomes and psychosocial well-being [15]. A great deal of research has documented the importance of a person-centered approaches in diabetes care, supporting the need for incorporating the cultural, social and psychological needs of T1D patients into patient education and clinical care [8, 15–18]. Empowerment is an effective model of person-centered education and care for people with T1D and may be particularly helpful for individuals with limited social and environmental resources [19–23].

In Brazil, almost 70% of T1D patients with low incomes receive their care in public tertiary care settings [12]. At State University of Campinas-Unicamp, the majority of T1D patients and their families have low incomes and are at high risk for developing clinical depression and/or DD. These patients experience considerable socio-economic stress and have few community resources to help them manage their disease.

In order to develop and implement an integrated psychosocial care program at Unicamp Diabetes Clinic that addresses the needs of T1D patients, we conducted a study to investigate the emotional burden of diabetes in this population. Our goal was to understand better the emotional side of T1D to help plan person-centered approaches for Brazilian T1D patients. The goals of this study were [1] to document the frequency of clinical depression, depression symptoms and DD, [2] to assess levels of empowerment, and [3] to determine the association between each of these measures and glycemic control in this low resource, Brazilian population of adults with T1D.

## Methods

This cross-sectional study included patients with T1D receiving outpatient care at the type 1 diabetes clinic of the University of Campinas tertiary care clinic.

Inclusion criteria were age 18 and older and diagnosis of T1D for at least 6 months. Exclusion criteria were cognitive impairment that could affect the patients’ ability to answer the protocol questions, history of major psychiatric disorders (such as schizophrenia, drug addiction, dementia), patients with severe diabetes-related complications (blindness, need for hemodialysis, limb amputations, and stroke) and pregnancy.

Patients were interviewed between December 2015 and December 2016 and were invited to take part in the study during routine consultations. Patients who consented to take part in the study gave permission for their clinical, laboratory and demographic data to be recorded. This study followed the principles of the Declaration of Helsinki and was approved by the University Ethics in Research Committee in December 2015 (CAAE number: 50864815.4.0000.5404).

The diagnosis of major depressive disorder (MDD) was made by the senior author based on the Diagnostic and Statistical Manual of Mental Disorders (DSM-5) of the American Psychiatric Association 2013 [24], in a face-to-face interview. Those diagnosed with MDD were so informed and were referred to the psychiatric service at Unicamp or to other psychiatric services in the community if they were not presently under care.

Evaluation of depressive symptoms was made with the depression subscale of the Hospital Anxiety and Depression Scale (HAD-D), developed by Zigmond et al. [25] and translated and validated into Portuguese by Botega et al. [26]. This scale was chosen because it does not involve somatic symptoms of depression, which could be confounded with symptoms of hyperglycemia. The HAD-D has 7 items and each is scored from 0 to 3. Bjelland et al. [27], through a systematic literature review, identified a cut-off point of 8 for clinically meaningful depressive symptoms [27].

The Diabetes Distress Scale (DDS) was used to assess DD. The DDS was developed by Polonsky et al. [28] and yields a total distress score and four subscales reflecting different sources of distress: emotional burden, physician-related distress, regimen related distress and interpersonal distress. The DDS has 17 items and utilizes a 6 point-Likert scale, in which the respondent indicates the presence of a problem for them, ranging from “not a problem” [1] to a “serious problem” [6]. Mean item scores of ≥ 2 are considered clinically meaningful [29]. This scale was validated in Brazil for the Portuguese language by Lima et al. [30].

Empowerment levels were measured by the Diabetes Empowerment Scale short form (DES-SF), developed by Anderson et al. [31]. DES-SF has 8 items, each rated on a 5 point Likert scale ranging from “strongly disagree” (1) to “strongly agree” (5). It was translated and validated in a Brazilian sample by Chaves et al. [32]. The DES-SF assesses psychosocial self-efficacy focusing on need for change, developing a plan, overcoming barriers, asking for support, supporting oneself, coping with emotion, motivating oneself, and making diabetes care choices appropriate for one’s priorities and circumstances [33].

Glycemic levels were evaluated by HbA1C, which was measured by high performance liquid chromatography (HPLC). The patient’s most recent lipid panel results were also recorded. Total cholesterol, HDL, LDL, VLDL and triglycerides were measured by commercially available enzymatic techniques.

Chronic microvascular complications of diabetes were assessed through chart review. Diabetic retinopathy was diagnosed based on fundoscopy examinations performed by the University Ophthalmology Department. Nephropathy was diagnosed if two or more urine samples separated by at least 30 days showed albuminuria results above 30 mg/g of creatinine. Neuropathy was diagnosed based on annual clinical examinations performed by the staff physicians at the diabetes clinic.

Seventy patients filled the inclusion criteria and were recruited for this study. Sixty-three patients responded to all the protocol scales. Statistic analysis was performed with these 63 patients. The descriptive analyses of the missing data were included.

### Statistical methods

Descriptive analyses were undertaken using means and medians, as appropriate. Differences between groups were assessed by the Mann–Whitney test for numerical variables, by the Chi Square test or by Fisher’s exact test for categorical variables, as appropriate. Non-parametric tests were used due to the sample size and non normal distribution of some variables. The association between two numerical variables was measured by Spearman’s correlation coefficient.

The association of MDD and other variables on glycemic control was assessed by linear and multivariate regression analysis, using stepwise criteria. All analyses were undertaken using SAS version 9.2 for Windows. Statistical significance was set at 0.05.

## Results

Of the 63 patients, 23 (36.5%) were male, 41.2% had a partner and 80.9% reported an income reaching until 3 Brazilian minimal wage [34]. Sixty two patients were using multiple daily injections and only one was using insulin pump. The total sample mean HbA1C was 10.0% (± 2.0%) (Table 1).

**Table 1:** Clinical, demographic and laboratorial characteristics of T1D patients

Variable (n = 63)	Mean	SD
Age	31.65	8.93
Years of T1D	16.05	8.57
Education	11.59	3.63
Number of glucose self-measures/day	3.00	2.70
Number of complications	1.00	1.13
HbA1c	10%	2%
Cholesterol	180.59	46.29
HDL	53.29	12.44
LDL	104.42	43.36
Triglycerides	103.74	72.65
VLDL	20.29	12.83

Variable	N (%)	N (%)
Gender	40 (63.49%) Female	23 (36.51%) Male
Income	51 (80.95%)—until 3 minimal Brazilian wage	12 (19.05%)—above 3 minimal Brazilian wage
Conjugal status	26 (41.27%)—have a partner	37 (58.73%)—don’t have a partner

Descriptive analyses: age (years), time of T1D (years), education (years), self-reported number of self glucose measures/day, Col, HDL, LDL, VLDL, triglycerides (mg/dL), sex (%), income, according IBGE

### Frequency of MDD, high depressive symptoms and high DD

In this low socioeconomic sample (SES) of T1D adults, the frequency of MDD was 34.9%, based on a structured interview using DSM-5 criteria. The frequency of relevant depressive symptoms was 34.9%, and the frequency of clinically meaningful DD was 57%.

### Relationship among MDD, depressive symptoms, DD, and empowerment

The patients with diagnosis of MDD reported significantly higher levels of depressive symptoms (*p* < *0.0001***)**. DDS total score was higher in those with MDD diagnosis (*p* = *0.0003*). Likewise the DDS subscale 1 (emotional burden), the DD subscale 3 (regimen distress) and the DDS subscale 4 (interpersonal distress) were higher in those with MDD diagnosis; respectively (*p* = *0.0033*), (*p* = *0.0026*) and (*p* = *0.0094*). The DDS subscale 2 was not different in those with or without MDD (0.43). The empowerment levels were not different in those with or without MDD diagnosis (p = 0.09).

Lastly, depressive symptoms, DD and empowerment levels were significantly intercorrelated (range r* = *− *0.31* to *r = *− *0.41*).

Women were more likely to receive the MDD diagnosis, but the difference was not significant (p = 0.09). Age did not discriminated between those with and without MDD (p = 0.8). The characteristics of patients with and without MDD are summarized in Table 2.

**Table 2:** Characteristics of patients with and without major depression disorder (MDD). Mann–Whitney test

	Patients with MDD (n = 22)	Patients without MDD (n = 41)	p-value
Age	31.32 ± 8.25	31.83 ± 9.37	0.88
Age at diagnosis	16.50 ± 8.06	14.68 ± 7.80	0.44
Education	11.45 ± 3.73	11.66 ± 3.62	0.76
Years of DM1	14.45 ± 8.57	16.90 ± 8.56	0.30
HbA1c	10% ± 3%	10% ± 2%	0.72
Number of complications	1.00 ± 1.08	1.00 ± 1.17	0.76
Gender: female	N (%)	N (%)	
	17 (77.27%)	23 (56.5%)	0.09

Age (years), age at diagnosis (years), education (years), HbA1c (%)

Table 3 shows the missing data supplementary analyses.

**Table 3:** Missing data descriptive analyses. Mann–Whitney test

	Variable	N	Mean	SD	p-value
No DES-SF (missings)	Age	7	26.43	7.00	0.14
	Education	7	11.14	4.14	0.19
	Years of DM1	7	16.00	6.81	0.96
	HADD	7	5.57	2.88	0.88
	DDS-total score	7	29.43	10.05	0.23
	DDS-subscale 1	7	10.14	4.95	0.35
	DDS-subscale 2	7	4.71	1.50	0.30
	DDS-subscale 3	7	11.43	6.00	0.30
	DDS-subscale 4	7	3.71	0.95	0.12
	HbA1c (%)	7	11%	3%	0.12
	MDD diagnosis: N (%)	2 (28.57%)	–	–	–
DESF-SF	Age	63	31.65	8.93	–
	Education	63	11.59	3.63	–
	Years of DM1	63	16.05	8.57	–
	HADD	63	6.87	5.27	–
	DDS-total score	63	40.21	20.27	–
	DDS-subscale 1	63	14.21	8.74	–
	DDS-subscale 2	63	6.49	4.40	–
	DDS-subscale 3	63	14.9	7.92	–
	DDS-subscale 4	63	6.35	4.25	–
	HbA1c (%)	63	10%	2%	–
	MDD diagnosis: N (%)	22 (34.9%)	–	–	–

Age (years), education (years)

*HADD* depression subscale of Hospital Depression and Anxiety Scale, *DDS-subscale 1* emotional burden, *DDS-subscale 2* physician distress, *DDS-subscale 3* regimen distress, *DDS-subscale 4* interpersonal distress, *MDD* major depression disorder, diagnosis according DSM-5 criteria

### Associations with HbA1C

Empowerment and high DD regimen distress were each significantly associated with HbA1C, according to the univariate linear analyses. Higher HbA1c was associated to lower empowerment levels (*p* = *0.01*) and higher DD regimen distress (*p* = *0.03*). These results are summarized in Table 4.

**Table 4:** Factors associated to HbA1c

Variable (N = 63)	Beta (parameter estimate)	CI (95%)	p-value
Female gender	− 2.43	− 12.80; 7.22	0.61
Education (years)	− 0.23	− 1.53; 1.06	0.71
Years of DM1	− 0.43	− 0.97; 0.10	0.11
MDD	− 1.75	− 11.50; 8.01	0.72
HADD	0.14	− 9.62; 9.90	0.97
*DES-SF*	− *1.11*	− *1.96*; −* 0.25*	0.01*
DDS-total score^a^	4.40	− 6.59; 15.38	0.42
DDS-total score^b^	7.58	− 3.78; 18.94	0.18
DDS-subscale 1^a^	1.18	− 9.36; 11.72	0.82
DDS-subscale 1^b^	− 6.01	− 18.22; 6.20	0.32
DDS-subscale 2^a^	− 2.43	− 17.44; 12.58	0.74
DDS-subscale 2^b^	− 4.00	− 19.01; 11.01	0.59
*DDS-subscale 3* ^a^	*11.14*	*0.95*; *21.33*	0.003*
DDS-subscale 3^b^	9.02	− 3.93; 21.98	0.16
DDS-subscale 4^a^	2.78	− 9.05; 14.60	0.64
DDS-subscale 4^b^	9.41	− 1.82; 20.65	0.09

Univariate linear regression

*MDD* major depression disorder, according DSM-5 criteria, *HADD* depression subscale of Hospital anxiety and depression scale, *DES-SF* Diabetes Empowerment Scale-short form, *DDS* Diabetes Distress Scale, *DDS-subscale 1* emotional burden, *DDS-subscale 2* physician distress, *DDS-subscale 3* regimen distress, *DDS-subscale 4* interpersonal distress

^a^DD > 2: distress clinically relevant (values between 2.0 and 2.9 = moderate DD; values > 3 = high DD)

^b^DD < 2 (low DD)

*p < 0.05

In multivariate analyses, empowerment levels were linked to glycemic control. Higher HbA1c was associated to lower empowerment levels (*beta* − *1.11*; *r-partial 0.09*; *p-value 0.0126*).

The presence of MDD diagnosis and high depressive symptoms were not associated with HbA1C in this expected direction (p = 0.72; p = 0.97, respectively).

## Discussion

The current study found a high frequency of MDD, high depressive symptoms, high DD, and low levels of empowerment in this population of low SES T1D adults seen in a tertiary care center in Brazil. The results indicate high depression rates and relatively high psychological distress in this population of T1D patients. These results are supported by other data in literature [1–3, 29].

This study also found a positive association between both empowerment levels, DD regimen distress and HbA1C. Low empowerment was significantly associated with high HbA1C. Also, high DD regiment distress was significantly associated with high HbA1C.

Patients with MDD reported higher levels of DD. The higher levels of DD in almost all subscales of DDS showed how the psychological diabetes distress seems to be challenging for the patients affected by MDD. The emotional burden, diabetes regimen distress and interpersonal distress are concerned areas for T1D patients with clinical depression in this population.

The significant intercorrelations among these psychosocial variables suggests how the emotional side of diabetes emerges as a major area of clinical concern among these low-resource adults with T1D. Depressive mood, high management distress and low levels of empowerment are known to be associated with low self-efficacy and feelings of hopelessness and burnout [35–39]. Lack of resources further reduces responsiveness of traditional educational intervention, and creates a cycle of sustained poor management with significant negative clinical consequences over time. The chronicity of T1D, the incessant nature of diabetes care and the demands for management provide a fertile environment for psychosocial problems among most individuals. For patients with low income, such as those enrolled in this study, this challenge can be even more disturbing.

The association between the psychosocial variables and glycemic control demonstrates further how high DD and lack of self-efficacy are tied to disease management and glycemic control. Their interaction over time is most likely circular, rather than linearly causative [40], as mood, self-efficacy and management affect each other over time.

How, then, should a comprehensive care facility that serves low SES T1D adults in tertiary public hospital settings like Unicamp respond to this clinical need? We suggest a two-stage process. First, the results of the current study reinforce the importance of psychosocial assessment in T1D clinical care. The DDS, for example, can provide educators and clinicians with a practical framework for identifying major sources of diabetes-related distress. The four DDS subscales enable the identification of specific sources of distress and allow for targeting specific interventions. Likewise, scores on HAD scale can quantify depressive symptoms and identify the patients at risk for MDD. Mitigating DD and properly addressing depressive symptoms may also facilitate patient engagement in self-management.

Secondly, our results emphasize that educators and clinicians should be trained to utilize strategies to improve empowerment levels of patients. A patient-centered approach provides an effective context to integrate the emotional, social, behavioral and clinical aspects of T1D into more effective and patient-relevant interventions. These strategies include reflection on self-management efforts and experiences, solving problems, identifying and addressing emotional and social problems, asking clinical questions and setting patient-determined behavioral goals [41]. Moreover, a specific focus on the social determinants of health can identify some of the major implications of poverty that affect diabetes management [12, 42, 43]. We suggest that diabetes educators and providers should be familiar with the concepts and implications of these important determinants of diabetes self-management. For example, at Unicamp clinic, the majority of patients use multiple injections of insulin and are provided with only 3 strips/day to monitor blood glucose levels. They have limited access to new technology, like continuous blood glucose sensors and insulin pumps. Other difficulties include the cost of medications, time away from work to receive care, and busy lives with children and family. Therefore, educational programs and care need to consider realistic, problem-based strategies, based on available resources rather than rely exclusively on traditional hospital or outpatient care protocols.

Two strategies, found effective in other low-resource clinical populations, should be considered. First, are peer support programs in which others with T1D are trained to assist patients with disease management, access to resources, and the emotional side of diabetes. The shared support of two individuals with T1D provides an effective medium for intervention and support [44–47]. This approach has been associated with improved self-care behaviors and metabolic control, decreased emotional burden and lower overall patient healthcare costs [44–46], making it cost efficient, in under-resourced clinical settings. Recent research has shown the feasibility of using diabetes educators to train and support peer leaders [47]. A peer support model increases the probability that empowerment-based interventions take place into communities. It also may decrease costs. This is a crucial aspect in underserved populations.

A second, complementary strategy includes the utilization of community health workers (CHWs). CHWs have been useful and effective in enhancing patient and community management of diabetes [46, 48–50,]. CHWs are trained to provide healthcare support and have a close understanding of communities they serve through shared ethnicity, culture, language, and life experiences. CHWs may help T1D patients by providing hands-on education, connecting them to community resources, and assisting them to better navigate into medical system [50]. This is specially beneficial in low resource settings, such as Brazil, where T1D patients face multiple challenges, as for example, lack of resources and the cost of transportation. Also, the roles played by CHWs in diabetes self-management are contextually and culturally appropriate.

Our findings have several limitations. First, the study is cross-sectional, such that the direction of causality among the variables included cannot be identified, especially the interaction of the emotional side of diabetes with glycemic control over time. Second, we were unable to assess specific aspects of diabetes management. It would have been helpful to assess the relationships among different aspects of the emotional side of diabetes with specific management tasks, e.g., number of missed boluses and frequency of hypoglycemic episodes. In addition the number of glucose self-monitoring was obtained by self-report. Finally, the results of this study are from a single public tertiary hospital in Brazil. It would have been helpful to evaluate other low SES T1D populations in other Brazilian regions.

## Conclusions

The findings of this current study are relevant to diabetes educators, clinicians and researchers who work with low-income T1D patients. The study showed high rates of MDD, depressive symptoms and DD, as well low levels of empowerment in this low SES population. Empowerment and diabetes regimen distress were linked to glycemic control. The results therefore support the need for professionals to prioritize patient psychological and psychosocial concerns when providing clinical education and care.

Focusing exclusively on blood glucose numbers to treat diabetes is incomplete, particularly for highly depressed and distressed populations. Comprehensive education and support for diabetes providers about the psychological and psychosocial aspects of care is crucial, especially in low-resource settings like those at Unicamp.

In addition, we suggest that the implementation of peer support and CHWs programs, focusing on supporting and sustaining patient self-care behaviors in community settings can be very beneficial. These approaches are cost-effective and are especially helpful with underserved populations.

## Abbreviations

T1D: type 1 diabetes
DD: diabetes related distress
MDD: major depression disorder
DSM-5: Diagnostic and Statistical Manual of Mental Disorders-5th edition
HAD-D: depression subscale of the Hospital Anxiety and Depression Scale
DDS: Diabetes Distress Scale
DES-SF: Diabetes Empowerment Scale short form
HbA1C: glycosylated hemoglobin
HPLC: high performance liquid chromatography
HDL: high-density lipoprotein
LDL: low-density lipoproteins
VLDL: very-low-density lipoprotein
BMI: Body Mass Index
SES: socioeconomic sample
CHW: community health worker
